# Does COVID-19 Vaccination Warrant the Classical Principle “*ofelein i mi vlaptin*”?

**DOI:** 10.3390/medicina57030253

**Published:** 2021-03-09

**Authors:** Michael Doulberis, Apostolis Papaefthymiou, Georgios Kotronis, Dimitra Gialamprinou, Elpidoforos S. Soteriades, Anthony Kyriakopoulos, Eleftherios Chatzimichael, Kyriaki Kafafyllidou, Christos Liatsos, Ioannis Chatzistefanou, Paul Anagnostis, Vitalii Semenin, Smaragda Ntona, Ioanna Gkolia, Dimitrios David Papazoglou, Nikolaos Tsinonis, Spyros Papamichos, Hristos Kirbas, Petros Zikos, Dionisios Niafas, Jannis Kountouras

**Affiliations:** 1 Second Medical Clinic, School of Medicine, Aristotle University of Thessaloniki, Ippokration Hospital, 54652 Thessaloniki, Greece; doulberis@gmail.com (M.D.); appapaef@hotmail.com (A.P.); spmedauth@yahoo.gr (S.P.); 2 Department of Gastroenterology, University Hospital of Larisa, Mezourlo, 41110 Larisa, Greece; 3 Department of Internal Medicine, General Hospital Aghios Pavlos of Thessaloniki, 55134 Thessaloniki, Greece; georg.kotronis@gmail.com; 4 Second Neonatal Department and NICU, Aristotle University of Thessaloniki, Papageorgiou General Hospital, 56403 Thessaloniki, Greece; gialamprinou@gmail.com; 5 Healthcare Management Program, School of Economics and Management, Open University of Cyprus, Nicosia 2252, Cyprus; elpidoforos.soteriades@ouc.ac.cy; 6 Department of Environmental Health, Environmental and Occupational Medicine and Epidemiology (EOME), Harvard T.H. Chan School of Public Health, Boston, MA 02115, USA; 7 Nasco AD Biotechnology Laboratory, Department of Research and Development, 18536 Piraeus, Greece; antkyriak@gmail.com; 8 Center for Integrative Psychiatry, Department of Psychiatry, Psychotherapy and Psychosomatics, Psychiatric University Hospital of Zurich, University of Zurich, 8032 Zurich, Switzerland; lechatzim@hotmail.com; 9 Department of Pediatrics, University Children’s Hospital of Zurich, 8032 Zurich, Switzerland; kikh.karafyllidou@gmail.com; 10 Department of Gastroenterology, 401 Army General Hospital of Athens, 11525 Athens, Greece; cliatsos@yahoo.com; 11 Department of Maxillofacial Surgery, 424 General Military Hospital, Ring Road Efkarpia, 56429 Thessaloniki, Greece; ichatzistefanou@usa.com; 12 ORL and Psychiatry Private Practice, 8032 Zurich, Switzerland; ap.melodos@gmail.com; 13 Neurology and Psychiatry Private Practice, 2502 Biel, Switzerland; semeninv@yahoo.com; 14 Alexandrovska University Hospital, Medical University Sofia, 1431 Sofia, Bulgaria; donsmaro@gmail.com; 15 Psychiatric Hospital of Thessaloniki, Stavroupolis, 56429 Thessaloniki, Greece; igkolia@yahoo.com; 16 Department of General, Visceral and Thoracic Surgery, Bürgerspital Solothurn, 4500 Solothurn, Switzerland; Dimitrios.papazoglou@spital.so.ch; 17 General Practitioner, Private Practice, 8032 Zurich, Switzerland; fc707@runbox.com; 18 Department of Nuclear Medicine, “Thegeneio” Cancer Hospital, 54007 Thessaloniki, Greece; kirbashristos@hotmail.com; 19 Department of Oral and Maxillofacial Surgery, Aristotle University of Thessaloniki, 54642 Thessaloniki, Greece; petroszikos@gmail.com; 20 FOB AKTION, 30002 Aktio, Greece; dionisisniafas@gmail.com

**Keywords:** COVID-19, SARS-COV-2, Hippocrates, *ofelein i mi vlaptin*, *Primum non nocere*, vaccine, vaccination

## Abstract

The current severe acute respiratory syndrome coronavirus 2 (SARS-CoV-2) pandemic warrants an imperative necessity for effective and safe vaccination, to restrain Coronavirus disease 2019 (COVID-19) including transmissibility, morbidity, and mortality. In this regard, intensive medical and biological research leading to the development of an arsenal of vaccines, albeit incomplete preconditioned evaluation, due to emergency. The subsequent scientific gap raises some concerns in the medical community and the general public. More specifically, the accelerated vaccine development downgraded the value of necessary pre-clinical studies to elicit medium- and long-term beneficial or harmful consequences. Previous experience and pathophysiological background of coronaviruses’ infections and vaccine technologies, combined with the global vaccines’ application, underlined the obligation of a cautious and qualitative approach, to illuminate potential vaccination-related adverse events. Moreover, the high SARS-CoV-2 mutation potential and the already aggregated genetical alterations provoke a rational vagueness and uncertainty concerning vaccines’ efficacy against dominant strains and the respective clinical immunity. This review critically summarizes existing evidence and queries regarding SARS-CoV-2 vaccines, to motivate scientists’ and clinicians’ interest for an optimal, individualized, and holistic management of this unprecedented pandemic.

## 1. Introduction

One of the most prominent and influential medical doctors of the classical antiquity is the Greek Hippocrates (Ιπποκράτης, 460–370 BC). His contribution, the so-called Hippocratic Corpus (1–65) covers a plethora of clinical practice fields as well as ethical principles, thereby establishing him as the father of western medicine [1,2,3]. In his book entitled “Epidemics” (Περί Επιδημιών, Bk. I, Section 5), among other critical parameters, he stated the following: “Practice two things in your dealings with disease: either help or do not harm (the patient)” *(*“*ἀσκεῖν περὶ τὰ νοσήματα δύο, ὠφελέειν ἢ μὴ βλάπτειν*”). Later, the also Greek physician Galen (Γαληνός, 129–216 AD) born in Pergamon of Roman Empire, incorporated the medical principles of Hippocrates and Aristotle and used only the second part of the Hippocrates’ quote, in Latin secondarily translated and widely known as “*Primum non nocere*” (First do no harm) [1,2,3]. 

Considering this fundamental health care prism, namely, “either help or do not harm the patient” *(ofelein i mi vlaptin),* we aimed to provide a state-of-the-art review of the relative literature regarding the potential benefits and concerns of currently available vaccines for the prevention of coronavirus disease 2019 (COVID-19).

Coronavirus disease 2019 (COVID-19) is an emerging infection first documented in Wuhan, China, in December 2019 and was declared officially as a pandemic in March 2020 [4,5,6,7]. COVID-19 is caused by the Betacoronavirus severe acute respiratory syndrome coronavirus 2 (SARS-CoV-2) a novel coronavirus lineage with RNA genome of 27–34 kb size with a characteristic surface spike-protein, the latter of which serving as one of its major pathogenetic components [4,5,6]. SARS-CoV-2 is regarded as an initially zoonotic coronavirus, displaying a genome overlapping of 88% with bat coronaviruses [8], which crossed species barriers to infect also humans [9]. According to the World Health Organization (28 December 2020) a total amount of 79,673,754 confirmed COVID-19 cases and 1,761,381 deaths have been documented globally [10]. In January 3, 2021, a total of 20,346,372 cases of COVID-19 and 349,246 related deaths have been reported in the US [11], while the global mortality rate of COVID-19 infection is estimated to be about 6% (5.7%) [12]. From the medical perspective, there is a demand to estimate inequalities in prevention and therapy, and long-term consequences of COVID-19 [13]. Since no established therapy have been approved against SARS-CoV-2 in humans, beyond other approaches, there is an urgent demand for a suitable vaccine to manage this emerging health issue. The global scientific community strives thereafter within an unprecedently intense and proactive battle to develop as fast as possible, effective, safe, and cost-effective vaccines along with antiviral treatments [4,14].

## 2. Brief History of Vaccines

One of the most distinguished advancements in the history of mankind is unequivocally the development of vaccines, which have contributed significantly to the amelioration of infections and improvement of health and longevity [15,16]. The very first developed vaccine is considered to be against smallpox developed by Benjamin Jesty as well as by Eduard Jenner in the 18th century [15]. It was based on a rather simplistic, albeit effective technique of attenuation of pathogens. Specifically, Eduard Jenner interpreted the results of Benjamin Jest in a more critical way and in 1798 demonstrated that variolation (inoculation namely of small amounts of a substance such as poison that induces immune or toxic effects) of zoonotic pox viruses (e.g., cowpox or horsepox) into human patients may be attenuated though provide immunity in case of future smallpox infection [15,17]. Etymologically, the word vaccine stems from the Latin word “*vacca*”, meaning cow [17]. Thanks to vaccination, the world is officially free of smallpox since 1980 [17]. 

Henceforth, a wide variety of attenuated vaccines have been developed. Among others, Louis Pasteur and his colleagues established the attenuation method and investigated numerous vaccines, such as the ones against *Pasteurella multocida*, anthrax, and rabies [15]. Additional well-known vaccines designed with the aforementioned method, include beyond others, against tuberculosis, poliomyelitis (polio), measles (oral), mumps, rubella, varicella, typhoid, and rotavirus [15]. A further vaccination method was the inactivation, namely, the inoculation of killed whole microorganisms. Typical paradigms are vaccines against rickettsia, pertussis, and polio (injected). A new revolutionary era was the in vitro development of vaccines by utilizing cell cultures [15]. Cell cultures enable the re-assortment of several RNA viruses, which is characterized by segmented genomes that through co-cultivation with clone selection by plaque formation leads to the isolation of viruses with mixed RNA segments. Further biotechnological achievements made it feasible to produce vaccines based on purified proteins or capsular polysaccharides; representative examples include diphtheria and tetanus toxoid, anthrax secreted proteins as well as meningococcus and pneumococcus polysaccharides [15].

The latest and “cutting-edge” technology for developing vaccines exploits the advances of genetic engineering. A prototype was the hepatitis B virus (HBV) vaccination, which made usage of the surface antigen; as yeast cells could synthesize in vitro large amounts of hepatitis B surface antigen [15]. Over the last decade an even more innovative biotechnological advancement allowed the production of mRNA vaccines, which may be applicable beyond infectious diseases to other diseases such as cancer. The very first report in vitro, however, dates back to 1990s [16,18]. Of note, a breakthrough study by Wolff et al. was published in *Science* as early as January 1990 [19]. The authors described the successful transfer of genes luciferase, β-galactosidase, and chloramphenicol acetyltransferase into murine muscle in vivo, by utilizing both RNA and DNA expression vectors. As closing remark, they deduced that “The intracellular expression of genes encoding antigens may provide alternative approaches to vaccine development.” To mRNA vaccines are thought to have several advantageous properties such us non-infectious agent, safe, efficient, stable, inexpensive and attractive for large-scale manufacturing [16,18].

Taken together, the vast majority of the scientific community and the general public do acknowledge the benefits of vaccines as a major civilization’s achievement and the designing of vaccine(s) against SARS-CoV-2, with proved efficacy without limitations mainly in terms of safety, are highly anticipated and required.

## 3. COVID-19 Vaccines

Although the immunopathology of coronaviruses is inadequately understood, the clarification of the molecular and cellular mechanisms behind the immune response triggered by SARS-CoV-2 will assist to develop vaccines and therapeutic strategies to control the infection and improve the clinical progression of patients. Since SARS-CoV-2 is a novel virus, the immune response elicited by this pathogen is not yet well defined. Within this emerging worldwide problem, characterized by high transmission rates and increased morbidity and mortality, a “pursuit for a protective holy grail” has been initiated [14,20]. The need for developing vaccines during the current pandemic represents a main challenge for science and medicine. Currently, the whole scientific community strives to develop vaccines with the aforementioned characteristics, which will be approved by both the global and local authorities and will become available for large-scale commercial usage. 

The previous accumulated experience from similar viruses such as SARS-CoV-1 and MERS-CoV (Middle East Respiratory Syndrome Coronavirus) is definitely of advantage [14,20]. 

Despite the generally long required waiting time typically more than 15 years to obtain the development of in a new vaccine, the abovementioned circumstances have particularly accelerated this issue. Thus, SARS-CoV-2 commercially accessible vaccines became available as soon as 15–18 months after the pandemic began [8]. In parallel with this effort, more than 140 candidate vaccines were in the pipeline by the middle of December 2020, whereas 15 of them have already reached a clinical phase III [8]; The latest WHO report, states that 152 vaccines prototypes are being tested in preclinical models, and 42 are tested in humans with ten of them in phase III [21].

Epigrammatically, the process of developing a vaccine consists of two key steps: (1) identifying an antigen and (2) developing a delivery approach for said antigen to achieve robust humoral and cellular immunity. These vaccine candidates can be categorized into four vaccine platforms: viral vector-, RNA-, DNA-, and protein-based vaccines [22]. Vaccines take typically at least 10–15 years to be developed owing to the phasing of vaccine development, from pre-clinical to clinical phases 1, 2, and 3, the latter being the conclusive efficacy trial. These phases are typically conducted sequentially, as they become increasingly costly. Therefore, before engaging resources to the following phase, it is essential to ensure that the data of the previous phase are convincingly sufficient to warrant further development. Anti-COVID-19 vaccine development has proceeded at an unprecedented pace, as many phases are conducted simultaneously because of massive financial resources poured into vaccine development. Within months rather than years, more than 30 vaccines have entered the clinical development pipeline, including a dozen in phase 2/3 trials. Concurrently, large-scale manufacturing was launched before data on safety and efficacy were collected to make safe and effective vaccines readily available.

In particular, the anti-COVID-19 vaccines being tested can be roughly classified into the following categories; (non) replicating viral vectors, virus-like particles, DNA platform, live-attenuated vaccines, inactivated vaccines, recombinant protein-based vaccines, plant based vaccines, and RNA platform [20]. Currently, at least five independent vaccines have been authorized for emergency use by different countries and authorities [23,24,25,26,27]; BNT162b2 based on an mRNA platform and developed by Pfizer-BioNTech, was the very first authorized SARS-CoV-2 vaccine for emergency usage in the US [23]; it has demonstrated, at least short-term, acceptable safety and efficiency [18,28]. Shortly after, a second approval has been received for mRNA-1273 vaccine by Moderna [26] by exhibiting similar characteristics in terms of safety and efficiency [29,30]. Gamaleya’s Sputnik V vaccine is viral-vectored and became wide known as the first registered anti-COVID-19 vaccine in the world (19 August 2020) [8,20,31,32] while it also recently obtained an authorization by the Russian Federation [27] after achieving an efficacy of more than 90% without any serious safety issues, as non-peer reviewed results claimed [8]. The fourth authorized for emergency use anti-COVID-19 vaccine with comparable technology to the Russian variant, is co-invented by Astrazeneca and Vaccitech of Oxford University [24]. An interim analysis of the four ongoing clinical trials [33] reported an encouraging short-term efficiency and an acceptable safety profile. A further vaccine and the first of its kind containing inactivated SARS-CoV-2 comes from the Republic of China and Sinopharm [25]. Regarding the latter yet unnamed vaccine, although no final results have been published, a clinical phase 1 as well an interim analysis yielded also comparable results [34,35]. A synopsis with the main characteristics of the previously described authorized for emergency use vaccines is illustrated in Table 1.

## 4. Raised Concerns of Current SARS-CoV-2 Vaccines

Following the two coronavirus vaccines which have been authorized for emergency use by US regulatory agencies, since there is an expectation for the development of many other vaccines in the immediate future, the scientific community as well as the general public are raising questions and concerns about the plethora of vaccines and their safety [36]; more than 190 vaccines are currently in development to inhibit infection by the novel SARS-CoV-2 [37]. What can be confusing, is that some trials have been withdrawn or put-on hold owing to safety reasons, while others have been declared to be effective and safe by Russian and Chinese authorities, despite not having passed rigorous efficacy trials [38,39]. Likewise, investigators and policymakers must consider how to deal with challenges that are unrelated to the vaccine candidates themselves. These comprise of vaccine hesitancy, weariness with current public health restrictions, and the staggering logistics of vaccinating the world’s population.

The high pace of work on vaccine development globally, and the premature application of an inadequately tested vaccine, could trigger the current vaccination fears. Therefore, each anti-SARS-CoV-2 vaccination program is positioned at risk, as already mentioned by some investigators [40]. Vaccination hesitancy, particularly in the context of an anti-COVID-19 vaccine, is a true global experience, and anti-vaccination actions are progressively increased [41,42]. Serious concerns may be raised concerning public participation in vaccination programs when the anti-COVID-19 vaccine is finally available globally, especially when considered the rising numbers of adults questioning the validity of preventive vaccination programs [43]. Important to note that, each individual’s autonomy is a fundamental value, and it does not justify, for instance, obligatory vaccination. Autonomy requires that individuals live according to rationally conceived plans of their healthiness and that the conditions for conceiving and pursuing these plans must be preserved. Individuals cannot voluntarily and irreversibly surrender their individual conditions necessary for autonomy. Regarding a conventional example, our global society prohibits voluntary slavery and dueling because enslaving or killing competent individuals who consent is incompatible with the conditions necessary for people to pursue their idea of a respected and healthy life [44,45]. John Stuart Mill argued that not all voluntary acts are justified by autonomy: “By selling himself for a slave, he abdicates his liberty; he forgoes any further use of it beyond that single act. It is not freedom to be allowed to alienate his freedom” [46]. Likewise, the adult’s freedom allows him to deny vaccination and obligatory vaccination by authorities obviously involves legal–judicial consequences.

In this issue several concerns emerging from the principle “*ofelein i mi vlaptein*” and the reported results of the abovementioned vaccines will be addressed. Further long-term safety and efficacy studies for additional authorized vaccines in the future will probably shed light to a plethora of, yet, unanswered issues. 

### 4.1. Coronavirus Family Vaccines and Autoimmunity

The occurrence of auto-inflammatory/autoimmune disorders in patients with COVID-19, such as autoimmune cytopenia, anti-phospholipid syndrome, Guillain-Barré syndrome or Kawasaki disease, requires attention for the development of new strategies for the management of life-threatening pathologies especially in critically ill patients [47]; an emerging body of evidence supports the connection between COVID-19 and autoimmunity [48,49,50]. Likewise, vaccine-induced autoimmunity from autoimmune cross-reactivity is associated with several pathologies including Guillain-Barré syndrome, multiple sclerosis, demyelinating neuropathies, systemic lupus erythematosus, narcolepsy, or postural orthostatic tachycardia syndrome in susceptible subgroups [51]. Therefore, one of the side effects of introducing a mass vaccination program could be an emergence of autoimmune diseases especially in individuals who are genetically prone for autoimmunity [52,53,54]. The composition of SARS-CoV-2 proteins and validation of similarities with human proteins are critical to predict an autoimmune response associated with immunity against host proteins and its clinical manifestations as well as potential adverse effects of vaccination [55]. In view of the evidence about the cross-reactivity of the SARS-CoV-2 proteins with human tissues and the probability of inducing autoimmunity reactions, exacerbating already morbid disorders, or leading to unpredicted pathologic events, it would only be prudent to do more extensive investigation concerning the autoimmune-inducing capability of the SARS-CoV-2 antigens [56]. The promotion and application of such an aggressive universal “immune passport” platform in the lack of thorough and accurate safety studies could lead to an enormous cost on the global community in the shape of another epidemic, this period a rising tide of increased autoimmune disorders and the years of suffering that come with them [56].

Finding a vaccine for a disease can normally take years [56]; as it was previously stressed, vaccine development requires typically 10–15 years or more [8] to be commercially available and they carry an estimated failure risk as high as 94% [57]. It is worth to be emphasized that both Middle East Respiratory Syndrome (MERS) and SARS-related research towards a successful vaccine did not yield any reliable vaccine after a decade of efforts [58].

These previous efforts for development of vaccines although included other coronaviruses distinct from SARS-CoV-2 and mRNA vaccines were not used, they worth to be briefly mentioned, since the accumulated experience may be useful for avoidance of vaccination risks against viruses with similar biological behavior. Regarding the vaccine against SARS-CoV, in an experimental study by Bolles et al. [59], young and one-year old mice were immunized with double inactivated SARS-CoV (DIV) vaccine. The authors revealed that the mentioned DIV provided only incomplete protection in this animal model and led to eosinophilic proinflammatory response in the lungs upon challenge.

Similar results were reported a year later by Tseng et al. [60], where vaccinated mice with SARS candidate vaccines it was shown that mice developed pulmonary eosinophilia with Th2 lymphocyte polarization [60]. Interestingly, autoimmunity has been shown to be characterized also by Th2-mediated eosinophilic clinical conditions [61]. Agrawal et al. reported a comparable vaccination risk in mice vaccinated with inactivated MERS coronavirus after an exposure challenge with live virus; murine lung parenchyma was infiltrated by eosinophils polynuclear cells with and upregulation of IL-5 an IL-13 [62]; eosinophilia has been associated with several autoimmune, rheumatologic and connective tissue disorders such as Churg-Strauss vasculitis, Wegener syndrome dermatomyositis, severe rheumatoid arthritis, sarcoidosis, progressive systemic sclerosis, systemic lupus erythematosus, IgG-4 related disease, and/or inflammatory bowel disease [63]. Another interesting relevant coronavirus is the coronavirus of cats, etiological agent for the feline infectious peritonitis (FIP). The vaccinations against FIP are reported to cause an enhancement of disease and it was suggested that feline patients being over-vaccinated and having parallel a predisposition to autoimmune disease may enhance the likelihood to develop an autoimmune disease [64]. As for human coronavirus strain 229E an autoimmune cross-reaction between myelin basic protein and T cells in multiple sclerosis has been previously reported [65].

It has been suggested that the phenomenon of molecular mimicry, namely, the sharing of homologous epitopes between virus and host, may lead to a cross reaction with autoantibodies production which could damage patients’ tissues. The cytokine profile seen in COVID-19 patients and the damage observed in the lungs resemble quite interstitial autoimmune diseases, compatible lesions of which have also been reported in necropsies of Chinese COVID-19 patients [48,49,50]. Furthermore, it has been reported that there is a heptapeptide sharing between viral spike glycoprotein and human proteome [49]. In the same context, further homology has been reported between soluble angiotensin converting enzyme (ACE) receptor 2 and SARS-CoV-2, which may also lead to cross-reaction and production of auto-autoantibodies; a similar pattern of lung injury related to COVID-19, one has already described for the scleroderma associated production of autoantibodies ACE-2 [66].

Closing this section, it is important to note, that the Centers for Disease Control and Prevention (CDC) underlines the missing safety data for this patient population [67]: “People with autoimmune conditions may receive an mRNA COVID-19 vaccine. However, they should be aware that no data are currently available on the safety of mRNA COVID-19 vaccines for them.” 

### 4.2. (COVID-19) Vaccines and the Road towards Final Approval; Preclinical Animal Models

There is a vigorous necessity for authentic COVID-19 animal models to enable the preclinical assessment of candidate vaccines and therapeutics [63]. Preclinical animal model-based studies constitute prerequisites during vaccines’ development to define the route of administration, immunological mechanisms, and the duration of obtained protection, in order to yield an optimal equilibrium between immunity and adverse events [68]; animal models offer help to elucidate the pathogenesis and mechanisms of SARS-CoV-2 disease biology as well as to clarify features of pharmacology, toxicology, and immunology of the therapeutics and vaccine strategies [69]. The first step of this process is to define the most appropriate animal model for the specific studied disease; infection route, receptors, mechanisms, and vulnerability should be similar to humans. Upon contaminated, the host should ideally develop respective clinical symptoms, signs and disease course, to provide adequate material to study the immune response [68]. In this regard, a safe timeline to ensure reliable results necessitates 9–10 years of preclinical studies, before proceeding to human administration [68].

The current SARS-CoV-2 emergency warranted a rapid recognition of animal models, parallelizing human COVID-19, to assist therapeutic management and vaccination against the increasing spread and mortality [70]. Among studied models, rodents, ferrets and nonhuman primates, especially Rhesus macaques, indicated promising results in simulating post-vaccination immune response and potential adverse results [71]. Nevertheless, Ehaideb et al. [72], by reviewing the adequacy of provided benefits, concluded that all models lagged behind an ideal in vivo pre-clinical substrate for SARS-CoV-2 vaccine development, as none of them replicated the severe or critical manifestations associated with death in humans with COVID-19. Furthermore, the common and severe complications, including acute respiratory distress syndrome and coagulopathy, were not recorded in those models, thereby signifying a further clinical limitation [73]. Concerning the Rhesus macaques being recruited for approved vaccines’ pre-clinical studies, vaccination stimulated neutralizing antibodies production, thus implying an overt indication of anti-viral protection [74]. In this experimental model, protective effectiveness is correlated with the titers of neutralizing antibodies against the spike protein though analyses of T-cell immunity are required [69]. SARS-CoV-2-infected macaques develop some lung pathology, but they do not show clinical manifestations of COVID-19 or death. The duration of clinically sufficient immunization or the potential adjuvant effect on a post-vaccination infection could not be assessed, due to the immediate innate viral clearance, the self-limiting pneumonitis without resulting in death and the aforementioned mild clinical course [72]. Moreover, relative experience with other viruses indicates that antibodies could augment inflammatory responses. This is termed antibody-dependent enhancement (ADE) of the disease, owing to the presence of poorly neutralizing cross-reactive antibodies that bind to the virus and enhance viral entry into cells [69]. This is a concern for SARS-CoV-2 vaccine development because host antiviral responses could become harmful post-vaccination [75]. Therefore, more comprehensive relative studies are warranted to estimate whether host responses to SARS-CoV-2 are protective or harmful. Considering the viral contamination, the previously mentioned ACE-2 and transmembrane serine protease 2 receptors, which possess high affinity to the viral particles and mediate their intra-cellular insertion, are differently distributed in human tissues compared to macaques [76]. Human type II pneumocytes, with high ACE-2 expression, consist primary targets of SARS-CoV-2, resulting in infection and respiratory damage. Nevertheless, non-human primates’ respective cells are characterized by lower ACE-2 expression, probably implying an endogenous resistance to COVID-19 infection compared with humans [77]. In this regard, a detailed assessment of animal models of COVID-19 and adherence to established time-line could ensure the avoidance of potential discrepancies and declination of a premature human administration.

Beyond model selection, the length of pre-clinical studies is the mainstay to evaluate medium- and long-term results of vaccination [68]. Although the current pandemic does not permit any delay, prolonged in vivo assessment would provide more evidence concerning the duration of anti-viral protection. Especially for RNA viruses, including SARS-CoV-2, repairing mechanisms do not recognize and restore mutations, thus resulting to their accumulation in viral genome. The altered RNA is transcripted to different protein structures-antigens, a process called "antigenic-drift". The novel antigens predispose to viral escape from immune surveillance, with influenza being the main representative; this is the reason of annual vaccination against this virus and emerging data imply a similar necessity for SARS-CoV-2, even for shorter intervals [68].

Apart from the therapeutic benefit, animal-based pre-clinical studies could elucidate potential safety concerns raised by previous CoV epidemics. More specifically, studies of SARS-CoV and MERS-CoV infections revealed an immune-enhanced disease manifested in two ways; an antibody mediated pulmonary disease or a defective protection of anti-CoV antibodies, which could facilitate a potential SARS-CoV-2 infection into target cells [78]. Based on previous studies, the mentioned ADE was considered to be mediated in case of Th2 stimulation after vaccination, and thus the evaluation of this eventuality in vivo was a prerequisite for SARS-CoV-2 vaccine administration in human studies [79]. The predominance of a Th1 response in SARS-CoV-2 vaccination was considered as a safe treaty to exclude a potential ADE in this emergent environment [78]. Nevertheless, except for the qualitative characteristics, the quantitative immune over-response could result in diseases’ development, thus resulting in immune Th-2 biased dysregulation [80,81,82]. Furthermore, the mentioned instability of viral RNA and the subsequent mutations could trigger an unpredictable ADE or insufficient anti-viral protection, predisposing to intense immune response upon post-vaccination infection with SARS-CoV-2, thus warranting a cautious approach in vaccine development. Nevertheless, a possible advantageous ADE should also be acknowledged, since evidence of cross protection between Japanese encephalitis and yellow fever has been previously reported [83].

To date, the most widely available SARS-CoV-2 vaccines are based on the mRNA technology, expressing the surface spike antigen [84,85]. Although previous studies reported encouraging results, some concerns regarding long-term safety have been interpreted over time [16]. Except for local and/or systematic immune response, which have been partially answered by the existing studies [82], the perpetual expression of mRNA-vaccine antigen, the toxicity of foreign nucleotides in host genome, and the induction of self-targeting antibodies required in vivo long-term evaluation before disposal in global community, to identify, at least, subpopulations at risk of autoimmune diseases and provide other alternatives. Moreover, the presence of RNA in extracellular space seems to predispose to endothelial damage, intercellular conjunctions relaxation and edema, increased viscosity, hyper-coagulation and thromboembolic incidence [16].

Currently there is no evidence that any of the in vitro or animal models of coronavirus infection consistently predict the experience in humans. Although animal models of SARS-CoV-2 infection may clarify immune protection mechanisms, future relative remarks of augmented disease in people receiving candidate vaccines for COVID-19 are mandatory to recognize the risk of disease immune enhancement [82].

To conclude, bypassing the established process with animal model studies gave rise to some substantial queries concerning prolonged efficacy and safety of novel vaccinating techniques and formulations against SARS-CoV-2. The disposal of vaccines in global population necessitates institutionalized approach, based on well-designed studies to yield the optimal preventive results, lacking complications.

### 4.3. Coronavirus Family Vaccines and Adjuvant Toxicity

The natural history of vaccine evolution led gradually to a reduction of immunogenicity through a swift from whole attenuated/killed microorganisms to some purified subunits or components of them. The development of vaccine adjuvants offered a promising solution by, among others reducing required doses and potentiating vaccine efficacy with adequate immune response [86]. Among the plethora of used adjuvants, for the SARS-CoV, MERS, and SARS-CoV-2 preclinical and clinical trials the most studies adjuvants include aluminum salts (alum), emulsions, and Toll-Like-Receptors (TLR) agonists [87]. Regarding the first category, the previously mentioned work by Bolle et al. [59] revealed that aged mice vaccinated with DIV plus alum had high levels of pulmonary eosinophils upon subsequent challenge. The lung eosinophilia was estimated through histopathology and flow cytometry. In detail, they detected an increase of the eosinophil chemoattractant CCL11 (eotaxin) at 2 and 4 days after the infection (*p* < 0.01). Tseng et al. [60] reported compatible results; vaccinated mice with SARS candidate vaccines with or without alum adjuvant developed pulmonary eosinophilia with Th2 lymphocyte polarization [60]. However, further studies implicating SARS-CoV-2 vaccines and alum without available toxicity information or being reported as safe should equally be acknowledged [87]. As far as the mRNA COVID-19 vaccines are concerned, although they do not contain any of the classical adjuvants, they include, however, for first time in a vaccine lipid nanoparticles, which serve as delivery vehicle and considered as an adjuvant. The lipid nanoparticles are PEG-lated, namely, conjugated with polyethylene glycol, the last of which have been accused for, rather rare, albeit life threatening anaphylactic reactions, if not promptly recognized and appropriate treated [88]. Interestingly, in preclinical animal models of Crigler–Najjar syndrome some mRNA toxicity of hepatic parenchyma has been reported when delivered in lipid nanoparticles. Moreover, although rabies mRNA used in a clinical human trial was considered to be generally “safe” the reported self-limited systemic and local adverse effects may sketch the inflammatory potential and toxicity dynamics of mRNA vaccines, effects which have not been described to such an extent with DNA plasmids [89]. Noteworthy, mRNA is nowadays not considered anymore as immunologically inert. On the contrary, it is known that mRNA. along with DNA vectors. do activate the pleiotropic innate immune system, among others by activation of TLR3, TLR7, and TLR8 [89]. 

### 4.4. Transmission and Long-Term Efficacy/Side-Effects of SARS-CoV-2 Vaccinated People

There is no vaccine or medication that is totally free from adverse events or risk of complications. A list of adverse events of particular interest (known as adverse events of special interest (AESI)) through all body systems, including cardiovascular, neurological, immunological, musculoskeletal, and dermatological manifestations, have been agreed by the Brighton Collaboration in conjunction with Coalition for Epidemic Preparedness Innovations (CEPI), and the WHO with input from regulatory agencies and additional experts, as have the related case definitions and surveillance strategies [90,91]. Among others, listed adverse conditions include anaphylaxis, vasculitis, myocarditis, generalized convulsions, and meningoencephalitis.

Well-known mild side effects include local edema, redness, and pain at the site of injection as well as fatigue and transient [92]. However, there are also other potential adverse events such as allergic reactions [93], including the mentioned anaphylaxis, an acute and potentially life-threatening allergic reaction, that has been reported following SARS-CoV-2 vaccination, i.e., receipt of the first dose of Pfizer–BioNTech anti-COVID-19 vaccine during December 2020 [11]. The CDC has issued updated interim considerations for organizing the potential management of anaphylaxis [93]. Moreover, screening for contraindications and precautions prior to administering COVID-19 vaccines is required, while vaccine locations should have the required supplies available to treat anaphylaxis, should implement post-vaccination observation periods, and should immediately treat people presenting anaphylaxis signs and symptoms by intramuscular injection of epinephrine [93]; anaphylaxis can lead to death if not treated promptly [94]. Of note, CDC and the Food and Drug Administration (FDA) have received notifications of supposed anaphylaxis cases via several channels, including direct outreach by health care providers and public health officials and reports to VAERS (Vaccine Adverse Event Reporting System), the national passive surveillance (spontaneous reporting) system for adverse incidents after immunization, that is jointly operated by CDC and FDA [95]. 

Likewise, there are additional vaccination-related severe adverse events, and their long-term safety is of great significance and should be carefully examined before such a medication is released to the market. Vaccine-mediated disease enhancement is another example of major side effect: In this condition, the humoral immune response driven by a SARS-CoV-2 vaccine could facilitate the virus acquisition or even make the disease evolve more severely [82]. The aforementioned ADE or the enhanced respiratory disease (ERD) are typical examples of such an overexpression of the immune response to the viral [96]. A more thorough search of the relevant literature reveals several paradigms of how vaccination entails a risk of an immune system overactivation. The formalin-inactivated vaccine applied against Respiratory Syncytial Virus (RSV, FIRSV vaccine) over 50 years ago in the context of clinical trials significantly raised hospitalization rates for RSV in children vaccinated with FIRSV from 6 to 11 months of age. Moreover, two deaths were documented in the same age group. This vaccine-associated enhanced disease led to discontinuation of these clinical trials and, there is always a possible risk of such adverse event in the case of SARS-CoV-2 vaccines, since the very recent clinical trials were too short in duration and may have failed to detect disease enhancement as yet [82]. Recently, disease enhancement post vaccination was also noted with multiple adverse events to recipients and was considered as the cause of more than 500 deaths in Philippines until August 2019, mainly in children of school age who were Dengue-seronegative [97]. 

The induction of innate immune responses can possibly contribute, in a minority of patients, to an excessive elicitation of inflammation and subsequent tissue insult. This can be especially seen in elderly people in whom there is an underlying state of chronic low-grade inflammation called inflammaging [98]. Inflammaging is thought to be partly linked to alterations in gut microbiota along ageing [99]. The above arises concerns regarding the safety of initiating the SARS-CoV-2 vaccination program from the elderly population without sufficient data from long-term clinical studies in this specific age group. 

With regard to the current SARS-CoV-2 vaccines data, safety issues were raised on September 2020 after an observation of a case of transverse myelitis in an AstraZeneca–Oxford University Phase 3 clinical trial participant (AZD 1222). This adverse event led to temporary discontinuation of the trial [100]. Meanwhile, AstraZeneca vaccine confronts resistance in Europe since French health workers suffer strong side-effects, more than their mRNA commercial counterparts [101].

As for Moderna mRNA-1273 vaccine, the frequency of unsolicited adverse events, and serious adverse events reported during the 28 days after injection was generally similar among participants in the two groups [29]. However, it deserves to be mentioned, that the few cases observed with Bell’s palsy might not be a coincidence, as the authors stressed that; “The anecdotal finding of a slight excess of Bell’s palsy in this trial and in the BNT162b2 vaccine trial arouses concern that it may be more than a chance event, and the possibility bears close monitoring” [29].

Another safety concern pertains to patients with rheumatological and other autoimmune diseases who appear to be high-risk patients, and by receiving immunosuppressive agents can affect the efficacy of vaccination. Of note, no phase 3 COVID-19 vaccine trial included such population. Specifically, anti-CD20 therapies, mainly Rituximab, evoke prompt and longstanding B-cell wane. It is noteworthy that this depletion lasts for several months and normal levels are observed 9–12 months post administration of anti-CD20 treatment [102]. Taken that these medications also cause impaired humoral immune activation post vaccination, it is recommended that physicians should advise patients to have vaccination after waiting for at least six months post Rituximab administration [103]. Similar data have also suggested an insufficient immune responsiveness in patients treated with Ocrelizumab [104]. Both Ocrelizumab and Rituximab trials data (NCT02545868, NCT00676715) have suggested that protective neutralizing antibody and vaccination responses are likely to be mitigated until the emergence of new specific B-cells [104]. As an example of the predicted poor response to vaccination of individuals treated with anti-CD20, patients with Neuromyelitis Optica Spectrum Disorder (NMOSD) have been found to have a reduced titer and seroconversion rate (37.5 versus 75.0% healthy controls) post vaccination against influenza (H1N1) virus 3–5 weeks after therapy with rituximab [105]. 

In addition, the ethics of testing vaccines in pregnant women have been debated for several years [106], and, as in the case of above mentioned rheumatological patients, pregnant women were excluded from the initial phase 3 clinical trials of COVID-19 vaccines [107]. Pregnant women with COVID-19 are more likely to require mechanical ventilation than nonpregnant women [108], and women infected with SARS-CoV-2 during pregnancy are at a higher risk for preterm birth [108], thereby potentially requiring vaccination, the following concerns put into question the safety of initiating the SARS-CoV-2 vaccination program in pregnancy: Crucial gaps in knowledge data from German and US trials of the Pfizer–BioNtech mRNA vaccine (BNT162b2) indicate a broad immune response to the vaccine with induction of neutralizing antibody responses, helper T cell type 1 (Th1) CD4+ cells and expansion of effector memory CD8+ T cells in both men and non-pregnant women [109]. Whether an equal immunophenotypic profile is detected in pregnant women is currently unknown. However, these data raise concerns since successful pregnancy outcomes are greatly depended on heightened helper T cell type 2 (Th2) and regulatory T cell activity, with decreased Th1 responses [110]. Disruption of the balance of CD4+ T cell responses during pregnancy is related with adverse perinatal outcomes, including fetal loss and preterm birth [111]. Moreover, there are concerns that neonates born to mothers with altered CD4+ T cell responses may have long-term sequelae [112]. Therefore, pregnant women and their obstetricians will need to use available established data to weigh the benefits and risks of COVID-19 vaccines. Some issues to be estimated when counseling pregnant persons include relative data from animal studies, potential risks to pregnancy of vaccine reactogenicity or the pregnant women’s underlying high risk conditions [107].

A further vulnerable population of patients are those with liver pathologies; patients with cirrhosis are at higher risk of developing severe COVID-19 and worse liver-related consequences as compared to those with non-cirrhotic liver disease. In particular, patients with fibrotic liver disease and those with orthotopic liver transplantation would be considered as principal targets for prophylaxis of COVID-19, as all other highly susceptible patients [113]. According to a recent Lancet Gastroenterology article, the number of included patients with liver pathologies was extremely scarce, although the three leading clinical trials of phase 3 (Pfizer/BioNTech, Moderna, and the AstraZeneca/University of Oxford) recruited almost 100,000 individuals. For instance, in the Pfizer clinical trial, 217 participants were included (namely, 0.6%), out of whom only three were characterized by a moderate to severe disease [114]. 

Moreover, there is no available scientific evidence regarding the impact of mRNA COVID-19 vaccines on neurological patients with severe headaches such as migraines with(out) aura and cluster headaches. Since both mRNA vaccines report a quite elevated of headache side-effects, it is reasonable to speculate, that individuals with chronic and severe headaches including the aforementioned entities may be at greater risk for an exacerbation and modification of their threshold for onset of headaches, giving thus birth to a perpetuation and vicious circle. Additionally, any interaction with the new developed anti-CGRP (calcitonin gene related peptide) antibodies such as erenumab is still unknown.

The absence of sufficient animal studies stands as another drawback of the newly-developed SARS-CoV-2 vaccines, as it was stressed previously. Animal models are of high magnitude in selecting an antigen as a vaccine candidate. These non-human studies provide crucial information regarding the disease transmission, the length of protection as well as evidence on the triggered immune response [115]. As mentioned before, animal studies is considered the critical step before proceeding to research in humans. It is apparently easier to look into the trajectory of an infectious disease in animals as they can provide the research material for histopathological analyses. Moreover, animal studies can provide intimate information about contingent reservoirs of the virus in an organism [68].

The duration of the vaccination’s provided protection is also still under investigation. There is no long-term evidence; thus, only predictions can be done at this point. Moderna vaccine mRNA-1273 has shown to offer at least three months of elevated binding and neutralizing antibodies at the dose of 100 µg, though more results are expected [116]. Lumley et al., by recruiting 12,541 health care workers, reported that the presence of anti-nucleocapsid or anti-spike IgG antibodies was correlated with a significantly reduced risk of SARS-CoV-2 reinfection in the forthcoming six months [117]. There is no quick way to determine the length of immunity to the SARS-CoV-2 virus, and companies together with approving authorities will need to observe this carefully within the next several months [118].

Important to note that the FDA has given Emergency Use Authorization (EUA) to both authorized for emergency use mRNA SARS-CoV-2 vaccines as yet, placing them into strict long-term surveillance program to identify potential side effects and further investigate the duration of protection offered by the vaccinations [119]. On the same direction, European Medicines Agency (EMA) have granted conditional marketing authorization to Pfizer–BioNTech vaccine. This type of authorization is practically given to medications that cover unmet medical needs and are supported by less comprehensive clinical data than normally required. Therefore, more clinical trials’ data are expected for a period of two years. In addition, independent studies conducted by EU authorities will offer more information regarding the long term safety and potential risks of these medications [120].

There has also been noted a discrepancy between vaccine doses and reported efficacy. The Oxford–AstraZeneca analysis found a striking difference in efficacy depending on the amount of vaccine delivered to a participant. A regimen consisting of two full doses given a month apart seemed to be just 62% effective. Surprisingly, however, participants who received a lower amount of the vaccine in the first dose and then the full amount in the second dose were 90% less likely to develop COVID-19 than those in the placebo arm [121].

Another drawback of this emergency approval granted to SARS-CoV-2 vaccines is that there is pressure on developers to offer the immunization to trial participants who received a placebo treatment. Subsequently, the control group allegation would obfuscate the data concerning medium- and long-term outcomes; a biased comparison regarding safety, duration and clinical impact of immunity stimulation between vaccinated and controls lurks [121]. 

This latter point is of great significance, as vagueness exists concerning the transmission potential after vaccination [122]. Vaccines have shown promise in preventing disease symptoms. However, none has shown that it prevents infection altogether, or reduces the shedding of the virus [122]. The same issue was raised in the mRNA 1273 vaccine efficacy results published on 30 December 2020. The collected data were considered insufficient to estimate asymptomatic infection rates. Therefore, it is considered unclear whether vaccination protects from transmission of the virus (infectiousness) [29].

It was the pressure to develop a vaccine within a span of months that led to the almost bypassing of animal studies. Without acknowledging that novel laboratory techniques require less time as compared to traditional processes, it is of concern that production managers have reported feeling pressure for vaccine development [123]. This acceleration of vaccines development could potentially be pernicious in terms of finally producing a vaccine that offers only suboptimal effectiveness to selected individuals [124]. The fast-track process of vaccine development, which includes the mentioned separate trial phases overlapping each other, entails the risk of missing adverse effects that would otherwise be detected in a long-term study. Moreover, among patients with autoimmune diseases, malignancies, those with diabetes and/or obesity or elderly people, the absence of specific high-risk groups of individuals in the studied populations can potentially lead to unpredicted severe short- and long-term side effects post vaccination. Thus, there is still much work to be done to clearly estimate the efficacy of the SARS-CoV-2 vaccines in specific high-risk individuals [31,36]. Recently in Norway, by introducing the Pfizer–BioNTech vaccine against COVID-19, thirty three elderly patients died shortly after receiving the vaccine [125]. This raises crucial concerns against vaccine administration particularly in old age. 

Additional studies will provide information on how long protection lasts, how well the vaccine prevents from severe COVID-19, how well it protects the immunocompromised people, children, and pregnant women, and whether it prevents asymptomatic cases from transmitting the virus [120]. With careful planning and systematic evaluation of the social value and risks, it can be ethical to conduct some placebo-controlled trials of vaccine candidates for COVID-19 even after obtaining an efficacious vaccine. Doing so, could be essential to efficiently address a pandemic that is inducing so much harm worldwide [126].

### 4.5. SARS-CoV-2 Gene Mutations and Efficiency of Current Vaccines

A total of 149 mutations have been observed in 103 sequenced strains evolved in the initial stage of SARS-CoV-2 pandemic [127]. The greatest number of the derived mutations included 67.4% of synonymous mutations and 84.3% of non-synonymous mutations, displaying new origin or people growth [128]. Among identified mutations, 23 were seen in surface glycoprotein (S) protein, 1 in envelope protein (E), 2 from membrane glycoprotein (M), and 7 from nucleocapsid (N) protein. These were mapped with the reported vaccine candidates illuminating the possible propositions on worldwide vaccines [129]. Among structural proteins, spike (S), envelope (E), membrane (M), and nucleocapsid (N) proteins are important in inducing immune responses; [130,131] the mentioned spike protein is the main tool for virus entry into the cells, which interacts with the host cell receptor ACE2; and several vaccine candidates focus on the spike protein, as it is targeted by neutralizing antibodies and plays a crucial role in viral entry [132]. In this regard, pathogens including viruses such as COVID-19 are constantly evolving owing to the processes of antigenic shift and drift. Such viral evolution leads to the occurrence of new serotypes (serovars) that prove deadly for humans-like influenza pandemics and SARS-CoV-2 is the recent example [133]. Both antigenic drift and antigenic shift induce changes in the antigenic properties of pathogens, rendering them unrecognizable by the host immune system. Thus, the viruses escape the immune surveillance of the host and are able to cause infection. Specifically, antigenic drift, as aforementioned, consists of a baseline characteristic of RNA viruses, providing an endogenous mutative potential, resulting in the absence of repairing processes, thus predisposing to epitopes’ alterations to evade antibodies binding [68]. Environmental suppressive conditions, such as host-to-host transmission and immune response, accumulate a sequence of nucleotide-amino acids-protein structure transformations reflecting to differences in viral phenotype and its interaction with host’s micro-environment [134]. In contrast to standard examples, such as influenza or human immunodeficiency virus (HIV), the SARS-CoV-2 expresses a 3′–5′ exonuclease, which functions as a guarantor of RNA stability, reproducibility and homogeneity through superintendence during genome replication [132,135]; viral diversity has challenged vaccine development efforts for HIV-1, influenza, or Dengue, though these viruses each constitute a more diverse population than SARS-CoV-2 viruses. In this regard, the mentioned surface spike (S) protein assists viral particles to attach and invade host cells and has been initially considered as relatively invariant, thus consisting a rational antibody target to yield immunity and host protection [136]. Therefore, the initially isolated SARS-CoV-2 genome (Wuhan-Hu-1), in December 2019, was the matrix for various vaccine developments, such as mRNA-1273, BNTX BNT162b2, and Ad5-nCoV [137,138]. Nevertheless, despite the recent onset, nucleic alterations in the gene encoding S protein are recorded [139,140,141,142,143]. The mainstay mutation of S protein consists of a replacement of the amino acid aspartate (D) from glycine (G) at position 614, which is currently the dominant variant globally [144], possibly through its durability in environmental stressors, as implied by its higher titer in studies with pseudotyped virions [142]. The mutational pattern of the 614 position follows a geographical distribution, with G614 predominating in West and D614 in East. The receptor-binding domain (RBD) of subunit S1 specifically interacts with ACE2, while the rest of the S protein does not. The RBD alone is enough to bind tightly to the peptidase domain of ACE2. Therefore, RBD appears to be a key determinant of virus–receptor interactions, virus–host range, tropism, and infectivity [145,146,147]. Although 614 locus is not included in the RBD, this mutation modifies the structure of S protein in a region that facilitates the protein’s binding to host cells surface receptors and the resultant fusion, thus impacting on an increased pathogenicity when compared to the primary virus [144,148]. In this regard, the mutated variant was hypothesized to trigger increased severity and mortality relative to defective immune response, with reformed G614 S protein remaining unliganded to immune system receptors/antibodies, as described with other viral infections such as respiratory syncytial virus (RSV) and HIV [144]. Nevertheless, studies in animal models with G614 infection revealed that the administration of serum, collected from infected subjects after wild-type viral infection, provided adequate neutralization of the mutated epitope [148,149]; and the commercially available mRNA-1273 induced CD8+ T-cell stimulation and antibody production to both 614 variants in pre-clinical studies, although the clinical protection is not translatable to human patients due to different COVID-19 related pathogenic mechanisms between mice and human [71,72,150]. G614-related infection yields, also, higher naso-pharyngeal viral copies and resultant infectivity, expressed by lower RT-PCR cycles [142], thus, probably, necessitating increased serum titer of neutralizing antibodies compared to D614; a complementary situation during SARS-CoV-1 pandemic was associated with ADE, in case of over-infection with the mutated virus [142,151]. Nevertheless, current data do not incriminate this variation for any ineffectiveness or adverse events of authorized for emergency use vaccines [151]. 

On the other hand, a plethora of additional mutations has already been recorded with some of them possessing a significant clinical and therapeutic impact. The most important region of S protein is the RBD sequence, due to its contiguity with epitope regions, and potential alterations could affect immune response and infection’s clinical outcome. In this regard, specific amino acid sequences, inside the S protein, with antigenic function have been recognized, such as 370–394, 450–469, 480–499, and 818–835 [152] and respective variations have been reported. Concerning clinically significant mutations, viral particles expressing A475V, L452R, V483A, N439K, Y508H, and F490L provided shielding against host humoral immunity [143] and studies investigating therapeutic options using monoclinic antibodies, revealed resistance of strains including the aforementioned mutations to some antibodies compared to controls [143]. The emerging British mutation is mainly characterized by N501Y variant and incorporates additionally 23 more mutations on the S protein, albeit with vague impact on immune response and vaccine effectiveness [153]. Moreover, the mentioned G614 mutation is accompanied with further genome instability and aggregation of mutations [134]. Specifically, in a plethora of countries, especially in the West, G614 variant included an additional mutation, ORF1ab 4715L, was associated with respectively increased mortality, while the co-existence of I472V provided increased infectivity and resistance to immune neutralization [143,154]. Taken together, immune-resistant variants should be closely monitored due to their potential to become dominant after eliminating current forms with vaccination.

In addition to amino-acid chain, glycosylation defines S protein’s stereotactic form and its interaction to other proteins receptors. Similar to previous CoV, current SARS-CoV-2 maintains a heavy glycosylation of S protein in 22 N-glycosylated regions, to regulate its relationship with immune reaction and virus–host interplay [155]. In this regard, genetic variability of glycosylation could affect basic viral functions, including its evasion from immune response [156]. Concerning infectivity, deficits in N331 and N343 glycosylation reduced viral colonization, whereas for resistance to antibody neutralization, N234Q variants were resistant and N165Q sensitive [143]. Regarding the immune-cells binding RBD, Serine 673, Threonine 678, and Serine 686 consist of three potential O-glycosylation sites, although homogeneity exists in the other N- or O-glycosylation sites. Those three sites are thought to protect viral particles from host’s defensive mechanisms, by creating a “mucin-like domain” [157,158,159]. The glycosylation may vary among host species and therefore vaccine development in bacteria, mammalian cells, and plant expression systems could affect antibody structure and effectivity in human patients [143].

Currently, several databases have been implicated to record RNA mutations of CoV-SARS-2 and assist scientists to design therapeutic and restrictive technologies. The Global Evaluation of SARS-CoV-2 Sequence (GESS) and GISAID databases consist continuously updated resources of COVID-19 detected mutations, which could provide evidence regarding stable, conserved epitopes of S protein chain to guide the development of universal, strain-independent vaccines [138,143,160].

The mutative potential of CoV-SARS-2, as an RNA virus, combined with the wild-type based emergent vaccine development, incubate the risks of inferior real-life results of massive vaccination and survival of neutralization resistant variants, thus necessitating further management. Further well-designed and mutation-based studies are necessary to recognize epitopes with low heterogeneity to yield optimal and long-term vaccination results. It is much too early to conclude that the virus has mutated into something more dangerous or more benevolent because the mutations can appear on a portion of the genome. The longer studying the virus, the more confidence unraveling.

### 4.6. Some Experts’ Concerns and Statements Regarding mRNA Technology Utilized for COVID-19 Vaccination

Worth-reporting are the concerns raised by some recognized experts, though others appear to avoid such rational claims. 

According to Professor Anthony Fauci, Director of the National Institute of Allergy and Infectious Diseases, Bethesda, Maryland, US, having already received the Moderna SARS-CoV-2 vaccine, there is no sufficient evidence, yet, that vaccines can completely stop transmission and replace social measures such as distancing and masking [161]. “In the worst-case scenario, you have people walking around feeling fine, but shedding virus everywhere,” says virologist Stephen Griffin at the University of Leeds, UK [122]. Dr. William Schaffner, a professor at the Vanderbilt University School of Medicine commented as following: “Even more so than usual, as we create vaccines, we’re sailing in uncharted water,” [162] and “New technology can sometimes cause unforeseen problems or side effects” commented Dr. Amesh Adalja, a senior scholar at the Johns Hopkins Center for Health Security [162]. Moreover, Norbert Pardi, et al. expressed also in a recent Nature State-of-the-art review [16] in favor of mRNA vaccines, their concerns in terms of efficacy. In particular, they stressed that the “ability of the vaccine to activate robust T follicular helper cell and germinal center B cell responses” is “an area that remains poorly understood.” The latter authors also commented, [16] that there is no regulatory guidance either by FDA or EMA to rule for mRNA as vaccine in humans [16]. Additionally, Stanley Plotkin, a vaccinologist and former pharmaceutical executive who currently consults for vaccine manufacturers stated in November 2020 as following: “personally, I’m waiting for further data concerning T-cell responses and duration of the antibodies” [163]. Sense was also made by the following statement of Professor of Virology in Michigan University, Oveta Fuller, one of the four specialists denying an FDA approval of the Pfizer mRNA vaccine: “The point is that if I say that here is the bar you need to reach which was 50% efficacy, over two months of the vaccine in people, Pfizer met that. It seemed like their study was set up to meet that at the minimum but it wasn’t set up to do a whole lot else” [164]. Finally, George Gao Fu, director of the Chinese Centre for Disease Control and Prevention, a health officer in China with particular impact, claimed that mRNA vaccines were being administered to healthy people for the first time and such a roll-out came with risks [165,166].

## 5. Conclusions

The way to manage diseases includes prophylaxis and treatment, with the first choice being most covetable. During the current SARS-CoV-2 pandemic, vaccine development created global optimism, especially in the absence of effective and established therapeutic regimens. Nevertheless, the scientific community should remain realistic and objective considering the research, production and approval process, effectiveness, and safety of available formulations. 

Emergent conditions, such as COVID-19, require rapid reflexes and decisive steps by health care scientists. On the other hand, when decisions and measures reflect to humanity and public health, no complacency and quality concessions are acceptable. Το bear the burden of duty and deal with current challenge successfully, medical professionals need to remember the compass-dictum of Hippocrates. 

## Figures and Tables

**Table 1:** Fundamental characteristics of the authorized SARS-CoV-2 vaccines.

Company	Vaccine Name	Technology	Efficacy	Origin	Participants	Stage
Pfizer–BioNTech	BNT162b2	mRNA	95%	USA/Germany	21,000 per arm	Phase 3 NCT04368728
Moderna	mRNA-1273	mRNA	94.1%	USA	14,000 per arm	Phase 3 NCT04470427
AstraZeneca–Oxford	AZD1222 (ChAdOx1)	AdenovirusNon replicating vector	70.4% (interim)	UK	20,000 vaccinated, 10,000 placebo	Phase 3 NCT04516746 (Ongoing)
Gamaleya	Sputnik V	AdenovirusNon replicating vector	91.4% (interim)	Russia	14,000 vaccinated, 4500 placebo	Phase 3, peer-review pending
Sinopharm	SARS-CoV-2 Vaccine	Inactivated whole virus (vero cell)	79% (interim)	China	More than 22,000 participants estimated	3 independent Phase 3 studies NCT04612972 ChiCTR2000034780, ChiCTR2000039000, peer-review pending
